# A chemical odyssey: Exploring renal stone diversity by age and sex in Punjab, Pakistan

**DOI:** 10.7555/JBR.38.20240039

**Published:** 2024-06-04

**Authors:** Muhammad Zubair, Rasool Zoha

**Affiliations:** Department of Pathology & Clinical Laboratories, Multan Institute of Kidney Diseases, Multan, Punjab 60000, Pakistan

Dear Editor,

Renal calculosis is one of the most common urological disorders worldwide, with a prevalence ranging from 1% to 13% across different regions^[1]^. Renal stones are crystal concretions that form on the inner surface of the kidney, resulting from disruptions in the metabolism, the excretion of stone constituents, or the formation of Randall's plaques and plugs. These stones are a result of various endogenous factors, such as age, sex, and genetic makeup as well as various exogenous factors like geography, weather conditions, and dietary factors^[2]^. Numerous epidemiological studies have documented variations in the incidence of renal calculosis geographically. These studies provide a piece of substantiating evidence for the proposition that individuals residing in warmer climates exhibit a greater lifetime prevalence of urolithiasis, attributable to the effects of dehydration^[3]^. Our country, Pakistan, located in the Afro-Asian stone-forming belt, has a prevalence of renal stones at approximately 16%^[4]^. The treatment of these stones, whether in the form of medical expulsive therapy or surgical intervention, leads to a significant burden on both hospitals and the economy.

Stone analysis plays a pivotal role in understanding of the underlying causes of renal calculosis, aiding in the formulating of treatment strategies and preventive measures. Both European and American urological societies recommend stone analysis at least once for each patient^[5–6]^. The present study aimed to analyze the chemical composition of renal stone disease in Punjab, Pakistan, and to determine the age- and sex-correlated prevalence of these stones.

For the present study, we retrieved data from the Nexus Pro Laboratory Information Management System of Chughtai Lab in Lahore, spanning a one-year period between July 2018 and June 2019. The lab received a total of 2956 stone specimens from Lahore and its neighboring cities. After pre-treatment to form pellets, all analyses were performed on the Perkin-Elmer Fourier Transformer Infrared Spectrophotometer (PerkinElmer Inc., Waltham, MA, US). Pure stones were characterized by a predominant component constituting 80%–100% of the composition, while mixed stones exhibited a major component comprising less than 80%. Patients who sought stone composition analysis from the laboratory, encompassing those with spontaneously passed or surgically removed urinary system stones, were included in the present study. Their urinary stone samples underwent chemical analysis upon receipt at the Clinical Biochemistry section. However, individuals whose stone chemical analysis data were either missing or incomplete were excluded from the present study.

The demographic information and continuous variables were presented as mean ± standard deviation. Categorical variables were described with frequencies and percentages. For the assessment of the significance of differences between sexes and age groups, the Chi-square test was employed. The statistical analysis was conducted using Microsoft Excel, version 13 (Microsoft Corporation, Redmond, WA, US), and IBM SPSS Statistics software, version 26 (IBM Corp., Armonk, NY, US).

**Figure 1:**
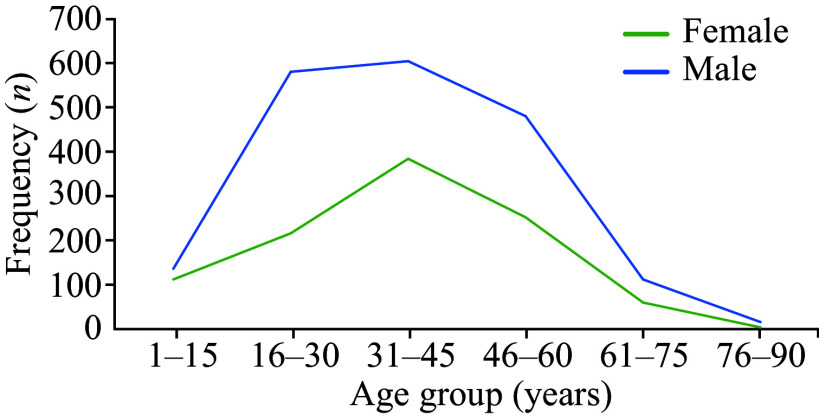
Age and sex distribution of renal stones in study participants.

Among the 2956 stone analysis results, 65.2% (1928) were males. The male-to-female ratio was 1.9∶1. The mean age of the study population was 36.83 (± 15.51) years, ranging from one to 90 years old. The chemical composition of all analyzed renal stones with their respective frequencies is shown in ***Table 1***. Calcium oxalate stones (pure) had the highest prevalence with their presence in 1335 patients. Pure stones had a higher rate of occurrence, compared with mixed stones. The prevalence of stones in males and females is shown in ***Fig. 1***, in which the male population showed a higher incidence of stones, compared with females in all age groups. However, their pattern of distribution across these age groups was the same and found to be statistically significant (*P* < 0.01). The patients aged 31–45 years had the highest incidence of renal stones with 33.4% of the reported cases, as compared with other age groups. However, the lowest incidence was observed in the 76–90 age group with only 0.7% of the whole study population.

**Table 1:** Chemical composition of renal stones in study participants

Type of stones	Number of the patients (*n*=2956)	Percentage (%)
Calcium oxalate (pure)	1335	45.16
Calcium oxalate (mix)	565	19.11
Uric acid (pure)	336	11.37
Uric acid (mix)	167	5.65
Carbonate apatite (pure)	223	7.54
Carbonate apatite (mix)	163	5.51
Ammonium urate (mix)	101	3.42
Struvite (mix)	46	1.56
Cystine (pure)	20	0.68

The outcomes derived from epidemiological investigations have broadened our comprehension of kidney stone diseases. Nevertheless, the analysis of stones is infrequently pursued, particularly in our geographic region, because of financial constraints and a prevailing skepticism regarding its utility. Currently, multiple modalities are available for stone analysis. However, we have chosen Fourier transform infrared spectroscopy because of its quick examination, moderate cost, and ability to identify organic components or non-crystalline substances.

Urinary stone composition varies worldwide with calcium oxalate stones identified as the predominant type in numerous studies^[7–8]^, including our investigation, where they accounted for a prevalence of 45.16%. Although the majority of the cases were generally considered idiopathic, urinary oxalate excretion played an important role in the formation of calcium oxalate stones, which was a continuous variable when correlated with the urinary stone risk in robust epidemiological cohort studies^[9]^. Additionally, the present study corroborates previous research, highlighting uric acid as the second most prevalent stone type and cystine stones as among the least common, consistent with findings from our geographic region^[10]^. This underscores the influence of diet and environmental factors on stone composition, which may further help us to study the mechanisms of urine stone formation and focus on preventative strategies.

Urinary stone disease not only directly affects individual well-being but also bears indirect consequences for the national economic scenario. Given that the highest rates of occurrence manifest among people with ages between 21 and 40 years, a subpopulation generally associated with an elevated economic productivity, the proficient management of the disease becomes particularly salient^[11]^. In the present study, the highest prevalence of renal stones was observed in the age group of 31–45 years, which is similar to a study showing the maximum frequency of patients between the 3^rd^ and the 5^th^ decade of life^[11]^.

In the present study, a higher preponderance of renal calculus disease was found in males than in females. The male-to-female ratio in the present study was 1.9∶1, which is consistent with similar studies done in our geographic region; for example, Bibi *et al*^[10]^ and Rafique *et al*^[12]^ reported the ratios of 3.8∶1 and 3∶1, respectively. Nonetheless, the ratios observed in other studies surpass those found in the present study, indicating a rising trend in the prevalence of urinary stones among women in recent years. This phenomenon may be attributed to the escalating rates of obesity or alterations in dietary patterns. The aforementioned observation aligns with the studies investigating the prevalence of the disease across sexes. These studies focus on the concentration of biomolecules present in the urine, which act as potent inhibitors of *in vitro* mineralization, demonstrating that the level of urinary inhibitors was three times higher in female than in male patients with renal stones^[13]^.

The present study possesses certain limitations. Firstly, it was a single-center cross-sectional design. Although the findings of the study are substantial on their own, additional prospective studies are required to yield definitive insights. Secondly, the study data were driven from a commercial lab where we had a limited access to the patient history, so the factors promoting the stone formation might not be delineated. Third, analyzer constraints at the time of the study prevented a detailed differentiation of mineralogical variants (*e.g.*, whewellite and weddellite) and a comprehensive reporting of mixed stone components.

In conclusion, the present retrospective cross-sectional study sheds light on the nuanced prevalence of kidney stone compositions in the population of Lahore and its surrounding areas, with an emphasis on the dominance of pure stones and the age group of 31–45 years. These results may have important implications for reducing economic burdens by guiding more efficient preventive and therapeutic strategies. Ultimately, this will contribute to an improved patient care and cost-effective healthcare outcomes.

Yours Sincerely,Muhammad Zubair, Zoha Rasool^✉^ Department of Pathology & Clinical Laboratories,Multan Institute of Kidney Diseases,Multan, Punjab 60000,Pakistan.^✉^Corresponding author: Zoha Rasool. E-mail: zoharasool97@gmail.com.
